# Label-Free Monitoring of Human IgG/Anti-IgG Recognition Using Bloch Surface Waves on 1D Photonic Crystals

**DOI:** 10.3390/bios8030071

**Published:** 2018-07-25

**Authors:** Alberto Sinibaldi, Agostino Occhicone, Peter Munzert, Norbert Danz, Frank Sonntag, Francesco Michelotti

**Affiliations:** 1 Department of Basic and Applied Science for Engineering SBAI, Sapienza University of Rome, 00161 Rome, Italy; agostino.occhicone@uniroma1.it (A.O.); francesco.michelotti@uniroma1.it (F.M.); 2 Fraunhofer Institute for Applied Optics and Precision Engineering IOF, Albert-Einstein-Str. 7, 07745 Jena, Germany; peter.munzert@iof.fraunhofer.de (P.M.); norbert.danz@iof.fraunhofer.de (N.D.); 3 Fraunhofer Institute for Material and Beam Technology IWS, Winterbergstr. 28, 01277 Dresden, Germany; frank.sonntag@iws.fraunhofer.de

**Keywords:** optical biosensors, one-dimensional photonic crystals, Bloch surface waves, human IgG screening

## Abstract

Optical biosensors based on one-dimensional photonic crystals sustaining Bloch surface waves are proposed to study antibody interactions and perform affinity studies. The presented approach utilizes two types of different antibodies anchored at the sensitive area of a photonic crystal-based biosensor. Such a strategy allows for creating two or more on-chip regions with different biochemical features as well as studying the binding kinetics of biomolecules in real time. In particular, the proposed detection system shows an estimated limit of detection for the target antibody (anti-human IgG) smaller than 0.19 nM (28 ng/mL), corresponding to a minimum surface mass coverage of 10.3 ng/cm^2^. Moreover, from the binding curves we successfully derived the equilibrium association and dissociation constants (K_A_ = 7.5 × 10^7^ M^−1^; K_D_ = 13.26 nM) of the human IgG–anti-human IgG interaction.

## 1. Introduction

In the field of proteomics, the study of the affinity of an antibody to its partners and the characterization of its specific binding is crucial to evaluating the biological performance of the recognition system itself. Indeed, specific protein determinations are becoming increasingly important clinical tools for therapeutics and for differential diagnosis of a number of disease states.

In this context, optical label-free bio-sensing is considered one of the most promising tools for the high-throughput detection of biomolecules. Optical biosensor platforms indeed provide reliable, rapid, quantitative, cheap and selective identification of biomolecules, which plays a crucial role in the clinical need for personalized treatment [1,2]. A label-free optical biosensor can assess the presence, activity and concentration of a specific analyte in a biological fluid; it can sense either a binding process (affinity ligand-based biosensor with the recognition element for a protein, peptide, ssDNA, RNA) [3,4,5,6] or a biocatalytic reaction (enzyme-based biosensor) [7].

Over the past few decades, due to their advantages of specificity, speed, portability, and low cost, the demand for optical biosensors that assay biomolecules quickly and accurately has increased dramatically for clinical applications [8] and especially for cancer diagnosis [9]. Among other label-free optical approaches [9,10,11], those based on the excitation of Bloch surface waves (BSW) at the surface of a dielectric one-dimensional photonic crystal (1DPC) [12] are demonstrated as a practical route to enhanced resolution and constitute an attractive alternative to surface plasmon polaritons (SPP)-based systems [13]. SPP-based biosensors exploit their evanescent field to probe the changes of the refractive index (RI) and interactions between immobilized receptors and analytes near a thin metal film [8]. Such a technique readily became a research hotspot in biosensing domain, and has gradually been commercialized. Similarly to SPP, BSW biochips make use of evanescent waves to sense RI and biological interactions as well. The unique properties of BSW, such as the small absorption of the dielectric materials and the wide range of tunability for the layer thicknesses to work at any wavelength, differ dramatically from SPP. Moreover, due to its intrinsic dielectric nature, the BSW biochips do not suffer from quenching of the fluorophores’ emission at the 1DPC surface in fluorescence applications [14].

The main purpose of this work is the development of BSW biochips operating in an angular interrogation scheme to demonstrate their use for studying a clinically relevant biological model such as human immune-gamma globulin (IgG)–anti-human IgG interaction [3].

The selected anti-human IgG (Fab-specific) antibodies, due to the reduced interspecies cross-reactivity to mouse or rat ascites fluids, are ideal for screening human monoclonal antibodies, as in the Ouchterlony double-diffusion test [15].

Therefore, the present technique has the potential to study antibody interactions by means of BSW on 1DPC with the possibility of extending to the determination of proteins in complex media such as protein extracts produced by hybridoma cells grown in vivo and in microphysiological systems in vitro [16].

## 2. Materials and Methods

### 2.1. 1DPC Optical Design and Fabrication

BSWs can be excited at the interface between a periodic dielectric multilayer, the 1DPC, and an external medium that in most cases represents the sample under investigation (aqueous solution). The BSWs are guided at the interface and the associated electromagnetic field decays exponentially in the 1DPC and in the homogeneous external medium [12,17]. Thus, the evanescent tail of the field can be used as a probe with a resolution in the order of hundred nm for studying biomolecular interactions at the sensor surface. The BSWs are excited by prism coupling in the so-called Kretschmann–Raether configuration [17] under total internal reflection (TIR) conditions. The angular reflectance spectrum shows a dip related to the excitation of a BSW, with a narrow width compared to SPP [18]. The resonance angular position depends on the optical properties of the external medium perturbed during the experiments.

The 1DPCs, for their periodical structure, are characterized by TE and TM photonic band structures (PBS) determining the unique property of 1DPC to work in both TE and TM polarization [12]. The 1DPC used in the experiments is formed, starting from the substrate, by a first silica matching layer, by repetitive units of tantala and silica and a top bilayer of titania and silica; this last layer of the stack is in contact with biological solutions (external liquid, EL) and consists of a functionalized SiO_2_ film. The complete structure can be synthetically sketched as follows: substrate/LHLHLH’L’/external medium, where L and H are materials with a low and high refractive index, respectively. The repetitive unit thicknesses are d_SiO2_ = 275 nm, for the L medium and d_Ta2O5_ = 120 nm for the H medium. The top bi-layer consists of a 20 nm thick titania (TiO_2_) layer (H’) and a 20 nm thick silica layer (L’). The materials have the following refractive index: n_SiO2_ = 1.474 + i5 × 10^−6^, n_Ta2O5_ = 2.108 + i5 × 10^−5^, n_TiO2_ = 2.28 + i1.6 × 10^−3^. The possibility of changing the materials and the geometry permits one to tune the main characteristics of the 1DPC, to allow for working in a wide wavelength range of operation.

**Figure 1:**
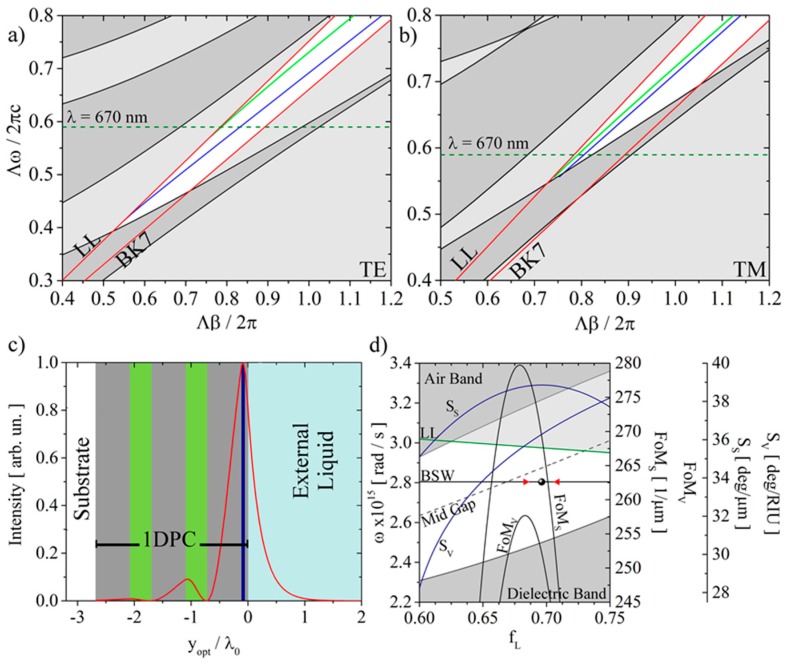
(**a**) TE and (**b**) TM photonic band structures for the silica and tantala 1DPC. (**c**) Electric field intensity (red line) into the 1DPC structure when a TE polarized BSW is excited. In gray are the silica layers, in green the tantala layers and in dark blue is the titania top layer. (**d**) TE band gap position as a function of the fill factor of the 1DPC periodic unit.

In Figure 1a,b, the calculated TE and TM photonic band structures are plotted for an infinite photonic crystal with a repetitive unit formed of a bilayer of silica and tantala and with period Λ = d_SiO2_ + d_Ta2O5_. Such numerical simulations were obtained by means of an iterative plane wave Eigen–Solver method [19]. The photonic band structures are plotted in the (ω, β) plane, where β is the transverse component of the wave vector and ω is the angular frequency. Normalization of the abscissa and ordinates by Λ guarantees the invariance of the band structure with respect to Λ provided that a constant ratio d_SiO2_/d_Ta2O5_ is maintained. In the plot, the permitted bands are represented by the dark gray shaded areas, while the light gray shaded regions are the forbidden states. The white regions, between the light lines for the external medium (LL) and the substrate (BK7), are the restricted portions of the forbidden bands where the excitation of a BSW can take place. Inside such white regions, the blue and green curves are the dispersions of the surface modes when the truncated 1DPC is either topped or not with the titania/silica bilayer, respectively. The dispersions were derived from the spectrally and angularly resolved reflectance, for excitation from the substrate side, calculated by means of a plane wave transfer matrix method (TMM) [20].

As shown in Figure 1a,b, the presence of a top dielectric load (TiO_2_/SiO_2_ bilayer) shifts the BSW dispersions towards larger β. For the TE polarization, this has the effect of bringing the dispersion to the center of the forbidden band (white region) and pushing the surface mode far from the LL, thus increasing the field localization of the BSW at the surface. At the operating wavelength λ, marked with a horizontal black line in Figure 1a,b, we therefore obtain two BSW, one TE and one TM. Figure 1c shows the normalized electric field profile of the TE polarized BSW, calculated by means of the TMM and plotted as a function of the normalized optical distance y_opt_/λ_0_, where λ_0_ = 670 nm. Such a normalized representation can be used to describe any 1DPC with the same optical thickness as the layers, independently from the working wavelength. The BSW optical decay length in the external medium, where the intensity is attenuated by a factor of 1/e with respect to the value at the 1DPC surface, is found to be 0.224, corresponding to a geometrical penetration depth L_P_ = 0.224 × λ_0_/n_EL_ = 113 nm, when n_EL_ = 1.33.

The 1DPCs used in the present work, which were designed to operate at a fixed ω_0_ = 2πc/λ_0_, are the result of an optimization procedure based on the analysis of the curves shown in Figure 1d. In Figure 1d, we show the shift of the TE forbidden band edges as a function of the fill factor $f_{L}=\frac{d_{{\mathrm{SiO}}_{2}}}{\Lambda }$, in the (ω, f_L_) plane. For each f_L_ value, we calculated the upper (ω_H_) and the lower (ω_H_) edges of the TE forbidden band at the BSW wave vector corresponding to ω_0_. The air and dielectric bands are dark gray, while the states above the LL (green line) are light gray. The BSW dispersion appears as a horizontal line at fixed ω_0_. The dashed line marks the mid gap, putting into evidence the distance from the BSW dispersion curve. The 1DPC used in this work is represented by a dot and the two red triangles are the ±3% limits of uncertainty deriving from the tolerance of the layers thicknesses that can be provided by the deposition technique, which is plasma ion-assisted evaporation (PIAD) under high vacuum conditions (APS904, Leybold Optics, Alzenau, Germany) [21].

In the present case, the optimization procedure was carried out by maximizing the surface sensitivity S_S_, which is defined as the change of the resonance position upon the addition of biolayer with thickness h = 1 nm and n_bio_ = 1.45 and is given in deg/nm [22]:

(1) $$S_{S}=\frac{d\theta }{\mathrm{dh}}=\frac{d\theta }{\mathrm{dn}}\frac{\mathrm{dn}}{\mathrm{dh}}=S_{V}\frac{\mathrm{dn}}{\mathrm{dh}}\approx S_{V}\frac{n_{\mathrm{bio}}-n_{H_{2}O}}{L_{P}},$$

where S_V_ is the bulk sensitivity defined as the change of the resonance position upon a change of the refractive index of the external medium given in deg/RIU. In particular, for the proposed 1DPC, the value of the bulk sensitivity is 31.8 deg/RIU.

From a practical point of view, the sensor performance is determined by the width W, depth D, and sensitivity S of the BSW dip observed in the reflectance profile (inset in Figure 1a). One can define a figure of merit (FoM) as [22]:

(2) $${\mathrm{FoM}}_{V/S}=S_{V/S}\frac{D}{W},$$

where the suffix (V or S) indicates that we are targeting either the surface or the volume sensitivity, respectively. The FoM permits us to estimate the sensor performances in terms of limit of detection (LoD):

(3) $$\mathrm{LoD}=\frac{\sigma }{S}=\frac{\alpha }{\mathrm{FoM}}\sqrt{\frac{\Delta \theta }{W\cdot N}},$$

where N is the number of CCD pixels on which the angular range ∆θ is sampled and α is a parameter that depends on the dynamic range of the detector [22]. So higher FoM values correspond to a lower LoD of the biosensor.

In Figure 1d, S and FoM are plotted as a function of f_L_. From such a diagram it is evident that FoM is maximum when the BSW resonance shifts closer to the middle of the forbidden band, indicating a sharper dip in the reflectance profiles. Moreover, the bulk sensitivity S_V_ is monotonically increasing when the resonance shifts closer to the LL because of the increase of the field penetration depth in the external medium. Finally, the surface sensitivity (S_S_) is at its maximum when using exactly the 1DPC structure designed, fabricated and used in the present experiments.

### 2.2. Description of the Optical Read-Out System

**Figure 2:**
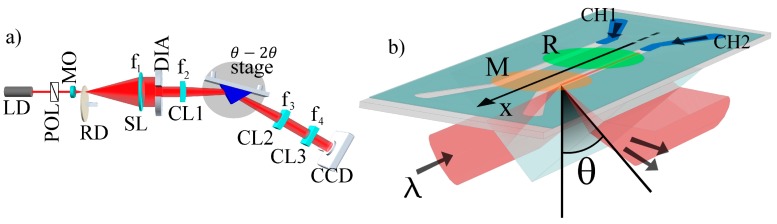
(**a**) Sketch of the optical read-out set-up. Pigtailed laser diode (LD); polarizer (POL); telescope system making use of a 40× microscope objective (MO), rotating diffuser (RD) and a spherical lens (SL, f_1_); diaphragm (DIA); cylindrical lens 1, 2 and 3 (CL1, CL2 and CL3 with f_2_, f_3_ and f_4_, respectively); CCD camera. (**b**) Prism and 1DPC in the Kretschmann–Raether configuration for the excitation of a BSW. The light, which carries angular components in the range Δθ, impinges on the 1DPC through a BK7 prism on a line along the fluidic cell. The output signal is recollected and analyzed by the CCD camera.

The optical read-out system, implementing an angularly resolved far field total internal reflection, is presented in Figure 2a. The light emitted by a temperature-stabilized (±0.01 °C) pigtailed laser diode (LD, Thorlabs, Newton, MA, USA, LPS-675-FC) at λ_0_ = 670 nm is collimated and linearly polarized (TE) with respect to the incidence plane by means of an input polarizer (POL). The laser beam is then expanded by means of a telescope. In the focal point of the telescope’s 40× microscope objective (MO), a rotating diffuser (RD) is destroying the spatial coherence of the beam. The beam is then collimated by the spherical lens (SL, f_1_) and focused by means of a cylindrical lens (CL1, f_2_ = 100 mm) onto the prism within an angular range Δθ ~3.8 deg. The θ–2θ rotation stage is used to set the average incidence angle (θ) around the resonance to be tracked; for the present 1DPC, that at θ = 68.763 deg when operating at λ_0_. The 1DPC biochip back facet is connected to the prism by means of a matching oil and the biochip is topped by a temperature-controlled (±0.01 °C) polydimethylsiloxane (PDMS) fluidic cell. The surface and volume of each channel are 63.5 mm^2^ and 12.7 μL, respectively. The microfluidic flow cell consists of a microscope glass slide with four connection holes and a structured adhesive spacer (Lohmann Adhesive Tape GL-187, Neuwied, Germany, thickness 200 μm) to define the two channels. The biological solution (sample vial) is injected in one of the two channels by means a motorized syringe pump generating a continuous sample flow in the microfluidics circuitry. The system is aligned in such a way that the beam illuminates a sharp line inside a fluidic channel and at the biosensor surface it is aligned perpendicularly to the incidence plane (Figure 2b). The reflected beam is then collected by a second cylindrical lens (CL2, f_3_ = 150 mm), which performs Fourier imaging on a CCD array detector. Therefore, each pixel of the CCD rows, lying in the incidence plane, corresponds to an angular component of the reflected beam.

A third cylindrical lens (CL3, f_4_ = 70 mm), rotated by 90 deg with respect to CL2, projects the sensor surface onto the CCD array detector; therefore, each pixel of the array columns corresponds to a position along the investigated region. This optical configuration sets the width of the angular detection range of 2.74 deg along the largest dimension of the CCD array (3388 pixel, 12.47 mm); therefore, one pixel corresponds to an angular width of 0.81 mdeg. In the other axis of the CCD array (2712 pixel), the position along the focused light strip in a 10 mm wide region is obtained.

### 2.3. Bioconjugation of the 1DPC Surface

The chemical modification of the biochip surface starts with cleaning by exposing the surface to a piranha solution (3:1 mixture of concentrated sulfuric acid and 30% hydrogen peroxide solution) for 10 min. Subsequently, the 1DPC were carefully rinsed with de-ionized (DI) water and dried under a stream of nitrogen gas. Once the hydroxyl covering of the surface was obtained, the biochips were immersed into a 2% (*v/v*) solution of APTES ((3-Aminopropyl)-triethoxysilane from Sigma-Aldrich, Darmstadt, Germany) in pure ethanol at ambient temperature (AT) for 1 h. Afterwards the biochips were removed from the silane solution, sonicated, rinsed with ethanol and dried under a stream of nitrogen gas. The APTES film was then stabilized by means a hot plate at 110 °C for 1 h.

In the present work, the bioconjugation strategy makes use of EDC (1-Ethyl-3-(3-dimethylaminopropyl)) carbodiimide (from Pierce, Waltham, MA, USA) to form an active EDC–antibody complex for the APTES-modified surface of the biochips [23,24].

To accomplish this, we prepared 5 μL of EDC (prepared in 0.1 M MES, pH 4.7) and mixed it with 495 μL of antibody (prepared in 10 mM sterile D-PBS 1X, pH 7.4). Moreover, the final pH of the crosslinking solution was close to the normal pH of 7.4, which is the most desirable pH to obtain an efficient immobilization of capture antibodies in immunoassays. The crosslinking solution was then incubated for 15 min at AT, which resulted in the binding of EDC to the carboxyl group of IgG antibody. The EDC-activated IgG antibodies were then allowed to react with the APTES-functionalized biochips, leading to the crosslinking of IgG antibodies to the free amino groups on the surface [24,25].

Afterwards, the EDC-activated antibodies were brought into contact with the sensitive surface. In one region of the sensing area, which will be referred to as the signal region (M in Figure 2b), we incubated Human IgG (IgG, I2511, from Sigma-Aldrich). In a second region, we incubated the same amount of anti-ovalbumin (Anti-Ova, 200-4133, from Tebu-Rockland, Le-Perray-en-Yvelines, France) to serve as a negative control or reference region (R in Figure 2b). Antibody coupling was stopped after 1 h interaction time at AT by washing away the residual solution. Finally, the whole biochip was immersed in 10 mg/mL of bovine serum albumin (BSA, A7906, from Sigma-Aldrich) overnight at 4 °C in order to block all residual reactive binding sites. As a result, capture antibodies were coupled covalently through Fc fragment by using such EDC activation procedure. Following such a bioconjugation strategy, capturing molecules retain their functional Fab sites, properly oriented for the specific detection of anti-human IgG (Fab-specific) antibody produced in goats (Anti-IgG, F5512, from Sigma-Aldrich). The whole set of reagents such as ethanol (99.8%), sulfuric acid (95%), 30% hydrogen peroxide solution and phosphate buffer saline (D-PBS 10X, pH 7.4) were obtained from Sigma-Aldrich and were used as received.

Once the biochip was mounted on the platform and the priming of the fluidic system was completed, the system was ready for antibody recognition experiments. Before starting the biosensing experiment, the surface was treated with a regeneration solution made of 10 mM glycine (from Sigma-Aldrich) and HCl with a pH of 2.0 (gly-HCl) for 10 min at AT [26]. Such a step improved the recovery of surface reactivity after the blocking step in BSA; in this way adlayers of molecules bound at the capturing surface could be easily removed from the covalently linked protein layers. As a further benefit, such a regeneration step permitted us to repeat the experiments on the same biochip, facing the variability introduced by the use of different BSW biochips and increasing repeatability [27]. The reusability of a biochip is a valuable advantage, even for disposable devices, considerably lowering the costs.

### 2.4. Data Format and Statistical Analysis

**Figure 3:**
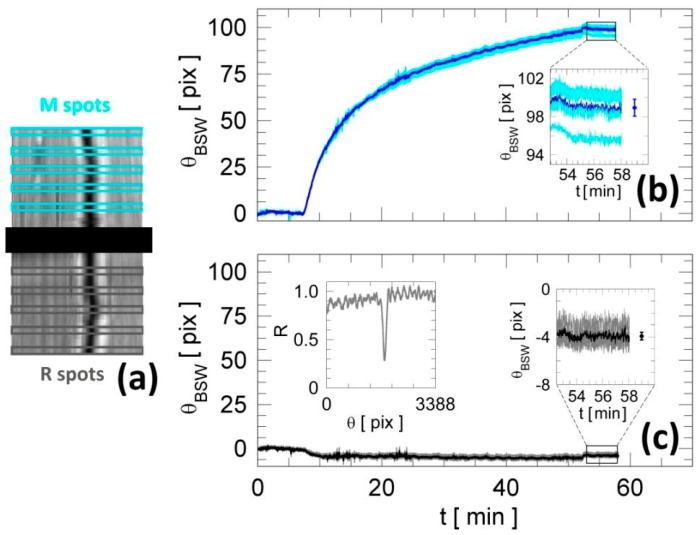
(**a**) 2D reflectance map through the CCD camera and definition of the five sensitive spots for both M and R regions. (**b**) Label-free angular spread on five adjacent spots in the measurements (IgG) and (**c**) reference regions (Anti-Ova) after analyte (Anti-IgG) interaction. The blue and black lines correspond to averaged curves from measurement and reference regions, respectively. In the zoomed areas, the points are the mean values with standard deviation for the five label-free residual angular shifts. In the inset of (**c**), an experimental reflectance is shown for one of the reference spots.

Figure 3a reports a typical CCD output of the platform during an assay. As previously mentioned, the surface of the biochip is divided into two regions in which two different capture antibodies are immobilized: measurement and reference. In turn, by tracking the minimum position θ_BSW_ (inset of Figure 3c) as a function of time, one can record sensograms for the two different regions. Figure 3b,c show the label-free resonance positions recorded during an exemplary assay in five different spots for the measurement (M, cyan curves) and the reference (R, gray curves) regions, respectively. Such an exemplary assay consists in the injection of Anti-IgG dissolved in D-PBS 1X (analyte: Anti-IgG). All curves in the measurement region (ligand: IgG) show a clear shift of the resonance angle due to the increase of surface mass density, which was not observed for the reference region (ligand: Anti-Ova). Each of these five spots is 100 rows of the CCD wide, corresponding to a region width of approximately 230 µm on the biochip surface. The averaged kinetic curves for the five adjacent areas, constituting the measurement (blue) and reference (black) responses, are also represented in Figure 3b,c. The errors were calculated as the standard deviation of the mean of the signals in the five spots in static conditions after the D-PBS 1X washing step (see the zoomed areas in Figure 3b,c). In order to normalize the signals, the average response recorded in the reference region was used to correct for drifts of the resonance position due to non-specific physical effects such as bulk refractive index changes, temperature fluctuations and pressure effects. Data analysis was performed by means of custom software developed in LabView, allowing us to handle the statistical analysis according to the procedure described above.

## 3. Results and Discussion

**Figure 4:**
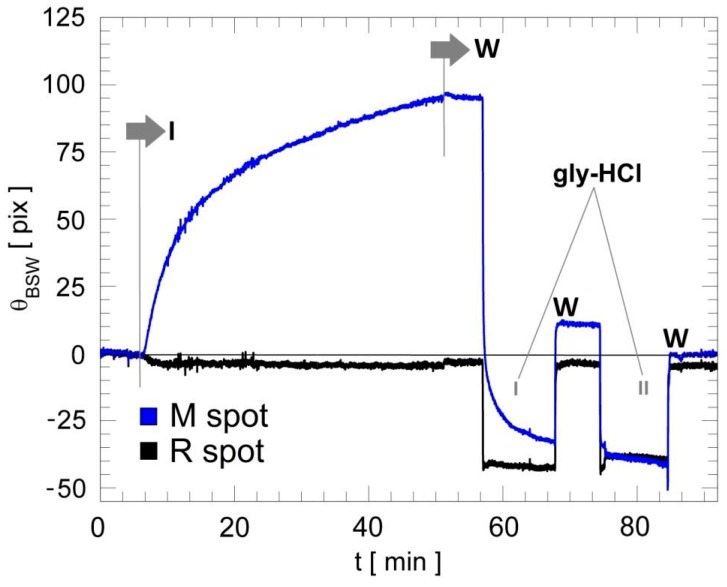
Sensograms of two spots in response to an Anti-IgG injection and two regeneration steps in sequence. The two sensograms correspond to the reference spot with anti-ovalbumin antibody (R, black curve) and the signal spot with human IgG antibody (M, blue curve). Then 10 min regeneration steps are performed to recover the starting conditions (before Anti-IgG injection, I marker). W indicates the washing steps.

In Figure 4 we show the angular shift θ_BSW_ in two spots inside the R and M regions measured in response to the injection of the target antibody Anti-IgG dissolved in D-PBS 1X. The injection of the Anti-IgG (I mark) is followed by a back and forth recirculation procedure to improve the reaction rate and avoid the formation of analyte-depleted volumes on top of the capturing regions. The M region, in which the human IgG is incubated, shows clear binding kinetics (blue curve). On the other hand, in the R region, no binding is observed (black curve), meaning that no interaction occurred between the immobilized Anti-ovalbumin and Anti-IgG antibodies. As for the first activation step, to properly regenerate the signal and reference regions, a solution of gly-HCl has been selected as the optimal solution for reactivation of the biochip surface [26]. These findings are clearly depicted in Figure 4. In fact, after the injection of the Anti-IgG and the related washing step in buffer (first W mark), two injections of gly-HCl solution were performed; already after two injections (10 min duration each), the sensograms recover almost completely to the starting signal level. At the end of the third regeneration (not shown in Figure 3), the two sensograms, both for signal (M) and reference (R) spots, show no drastic changes in the angular position of the resonance with respect to the starting levels at *t* = 0 min.

After the first regeneration step, only a partial recovery of the surface was produced for the measurement spot (e.g., Figure 4). A complete recovery of the surface was obtained after a second regeneration step, conferring comparable binding capacity, and thus reusability, to the biochips.

**Figure 5:**
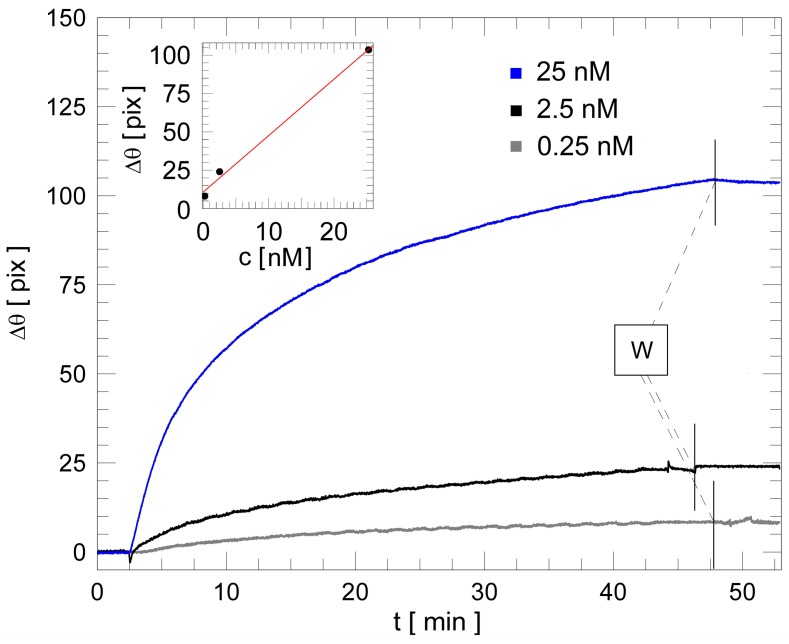
Series of increasing anti-IgG concentrations ranging from 0.25 nM (38 ng/mL, gray curve) to 25 nM (3800 ng/mL, blue curve). The washing steps (W) are marked with a vertical black segment. Inset: calibration curve obtained for different concentrations of Anti-IgG against human IgG immobilized receptors.

In order to obtain a calibration curve, the BSW biochips were tested with increasing concentrations of anti-IgG. In between each concentration, a regeneration procedure (consisting of three glycine-HCl injections) was performed. The result of such an iterative procedure is shown in Figure 5, in which the differential curves are shown (M minus R, ∆θ). For the sake of comparison, the sensograms were shifted temporally to align the anti-IgG injection points. The three concentrations were assayed sequentially on the same biochip, separated by the regeneration steps. The residual ∆θ of the differential curves obtained after washing (W in Figure 4) increases as a function of the concentration of the target antibody, which was 0.25, 2.5 and 25 nM, respectively. The slow oscillations observed during the binding reaction are due to the back and forth recirculation procedure. The residual angular shifts are considered after the washing in D-PBS 1X and waiting for a stable signal level in a buffer environment.

From the measurements shown in Figure 5, we can draw a linear calibration curve (inset of Figure 5) in the concentration range explored [28,29]. The slope of the calibration line is the surface sensitivity S_S_ with respect to the analyte concentration (measured in deg/M or equivalently in pix/nM) for the BSW biochips. Similarly to Equation (3), we can therefore evaluate an experimental LoD, with the assumption of linear dependence for c < 25 nM [29].

By taking into account the sensitivity *S_S_* and the standard deviation of the differential signal σ_diff_ = 0.7 pix (obtained from the standard deviations of the mean for both the M and R regions), the experimental LoD is found to be LoD = (0.19 ± 0.01) nM = (28 ± 1) ng/mL. By using Equation (3), it is also possible to quote the LoD of the present system in terms of minimum detectable refractive index change, LoD = 10^−5^ RIU. Such a value is therefore in the range of commercially available SPR platforms (LoD = 10^−6^ − 10^−5^ RIU). From the residual shifts ∆θ, obtained for the three different anti-IgG concentrations used in the assays, it is also possible to estimate the surface mass coverage and the surface density [30], which are reported in Table 1.

According to the LoD found, the minimum surface mass coverage for Anti-IgG detection is Σ_MIN_ = 10.3 ng/cm^2^ = 103 pg/mm^2^ and the minimum surface density is Γ_MIN_ = 68.4 × 10^9^/cm^2^ [30].

Nevertheless, due to the limited number of calibration points, we can only provide an estimated value for the LoD. Future investigations will be devoted to a rigorous analytical study of IgG–anti-IgG interaction in different biological matrices as well as in tissue samples [31,32]. In terms of sensing performance, the present technique can be compared with commercial platforms based on surface plasmon resonance (SPR) and electrochemical biosensors as well as with state-of-the-art optical fiber sensors. Indeed, our technique shows better performance with respect to SPR systems for physical [18] and biological [33] sensing parameters. Nevertheless, we are still far from the LoDs obtained by electrochemical [34] and nanocoated optical fiber biosensors [35].

From the label-free binding kinetics, it is also possible to extract association and dissociation rate constants (*k_ass_* and *k_diss_*) for the anti-IgG–IgG interaction. In particular, data related to the binding of the Anti-IgG in a buffer solution to the IgG immobilized onto the 1DPC solid-phase support show heterogeneous kinetics that can be modeled as follows [36,37]:

(4) $$R\;\left(t\right)=A_{1}\left(1-e^{-k_{on1}^{obs}t}\right)+\;A_{2}\left(1-e^{-k_{on2}^{obs}t}\right)+R_{0},$$

where $k_{on1}^{obs}$ and $k_{on2}^{obs}$ are the two observed on-rate constants, *A*_1_ and *A*_2_ are the corresponding amplitudes, and *R*_0_ is such that *R*(*t* = ∞) = *A*_1_ + *A*_2_ + *R*_0_.

Such a biphasic model indicates that more than one interaction is occurring and that the binding kinetics can be divided into two stages, an initial and a delayed interaction phase, in agreement with other interaction models [36].

**Figure 6:**
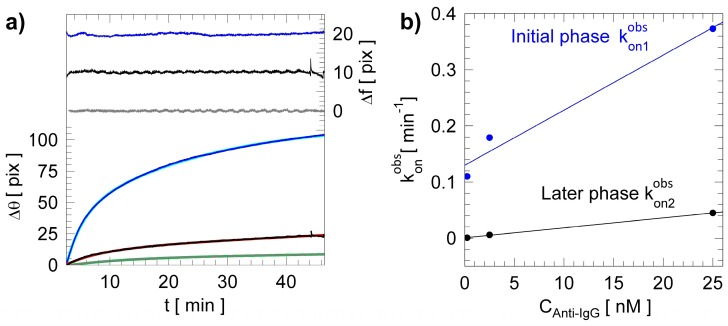
(**a**) Association binding curves for increasing concentration of Anti-IgG. On the right axis difference (∆f) between the biphasic fitting model (cyan, red and green) and experimental data (blue, black and gray), respectively, for 25 nM, 2.5 nM and 0.25 nM; (**b**) observed on-rate constants $k_{on1}^{obs}$ and $k_{on2}^{obs}$ for the initial (blue) and later (black) phases as a function of *C_Anti-IgG_*.

The bottom part of Figure 6a plots the experimental association phases and the related biphasic fitting models (cyan, red and green curves) for the three anti-IgG concentrations, respectively. The models are in very good agreement with the experimental data, as shown in the upper part of Figure 6a (right axis), where the difference between the experiments and the biphasic model (∆f) are reported. The variations are always below ±1.5 pix for all the anti-IgG test solutions assayed. The association *k_ass_* and dissociation *k_diss_* rate constants for the anti-IgG–IgG binding reaction can be indirectly obtained by plotting the two observed on-rate constants ($k_{on1}^{obs}$ and $k_{on2}^{obs}$) as a function of the anti-IgG concentration *C_Anti-IgG_* (expressed in nM). In fact, the $k_{on}^{obs}$ values vary with respect to *C_Anti-IgG_*, as follows [36,37,38,39,40]:

(5) $$k_{on}^{obs}=\;k_{diss}+C_{Anti-IgG}\cdot k_{ass}.$$

From such a linear dependency, we can determine the association rate constant *k_ass_* from the linear slope and the dissociation rate constant *k_diss_* from the intercept. Figure 6b shows the linear dependencies of the two apparent association constants, corresponding to the initial (blue) and later (black) phases of interaction, respectively.

From the curve fits, it is possible to extract, for the initial and delayed interaction phase, *k_ass_*_1_ = (1.60 ± 0.28)·10^5^ [M]^−1^ [s]^−1^, *k_diss_*_1_ = (2.16 ± 4.16)·10^−4^ [s]^−1^ and *k_ass_*_2_ = (0.28 ± 0.0065)·10^5^ [M]^−1^ [s]^−1^, *k_diss_*_2_ = (0.13 ± 0.009)·10^−4^ [s]^−1^, respectively. The determination of both *k_ass_* and *k_diss_* allows the calculation of the equilibrium dissociation and association constants K_D_ and K_A_, for both reaction phases.

However, a delayed interaction phase can reflect the presence of heterogeneous binding sites of the bound receptors, showing different affinities with the analyte or, alternatively, more than a single-step interaction [36]. In this latter case, a complex response can be triggered by an event after initial binding, e.g., a conformational change of the bound receptors, producing a sort of allosteric effect on the immobilized IgG. For these reasons, in the presence of biphasic kinetics, the faster rate constant (initial interaction phase) most closely describes binding events at the surface of the biochip. The slower rate constant (delayed interaction phase) is in general more complex and contains no readily interpretable kinetic information [36]. Thus, for the initial interaction phase, the equilibrium association and dissociation constants are K_A_ = 7.5 × 10^7^ M^−1^ and K_D_ = 13.26 nM. Such values relate to the results obtained by Chamiritski et al. in 2007 (K_A_ = 2.45 × 10^7^ M^−1^) for the same biological model [41]. Moreover, these findings are in agreement with the values found in the literature for the interaction of human IgG Fab fragments with goat anti-human IgG (Fab-specific), which showed K_D_ = 42 nM [42].

Further studies will be devoted to the investigation of the heterogeneous nature of the Anti-IgG–IgG interaction. BSW biochips, however, completely fulfill all the needs and requirements for binding affinity studies.

## 4. Conclusions

BSW technology has the potential to be a robust methodology for binding studies; in this case, a heterogeneous IgG–anti-IgG interaction is reported. The experimental results demonstrate the label-free detection of anti-IgG antibody based on BSW functionalized biochips. In the present work, the BSW biochips are designed fabricated in order to obtain the lowest LoD. The experimental LoD and Σ_MIN_ for nti-IgG antibody are found to be 0.19 nM (28 ng/mL) and 10.3 ng/cm^2^, respectively. In addition, from binding kinetics curves we successfully derived THE equilibrium association and dissociation constants (K_A_ = 7.5 × 10^7^ M^−1^; K_D_ = 13.26 nM) for the proposed biological model based on IgG–anti-IgG interaction.

Therefore, BSW biochips are a promising candidate for developing a class of novel label-free biosensors that can provide simultaneous measurement of RI and surface mass density as well as assess the binding affinity of the studied biological interactions. This enables easy operation, and sensitive, in situ and real-time label-free biosensing.

## Figures and Tables

**Table 1:** Experimental Anti-IgG concentrations, surface mass coverages and surface densities.

Protein	MW [10^3^ g/mol]	c [nM]	Σ[ng/cm^2^]	Γ[10^12^/cm^2^]
Anti-IgG	150	0.25	13.6	0.09
		2.5	38.5	0.25
		25	167.7	1.18
